# The *Shh*/*Gli3* gene regulatory network precedes the origin of paired fins and reveals the deep homology between distal fins and digits

**DOI:** 10.1073/pnas.2100575118

**Published:** 2021-11-08

**Authors:** Joaquín Letelier, Silvia Naranjo, Ismael Sospedra-Arrufat, Juan Ramón Martinez-Morales, Javier Lopez-Rios, Neil Shubin, José Luis Gómez-Skarmeta

**Affiliations:** ^a^Centro Andaluz de Biología del Desarrollo, Consejo Superior de Investigaciones Científicas, Universidad Pablo de Olavide, and Junta de Andalucía, Sevilla 41013, Spain;; ^b^Center for Integrative Biology, Facultad de Ciencias, Universidad Mayor, Santiago 8580745, Chile;; ^c^Department of Organismal Biology and Anatomy, University of Chicago, Chicago, IL 60637

**Keywords:** fin growth, Gli3, Shh, fin-to-limb transition

## Abstract

One of the central problems of vertebrate evolution is understanding the relationship among the distal portions of fins and limbs. Lacking comparable morphological markers of these regions in fish and tetrapods, these relationships have remained uncertain for the past century and a half. Here we show that Gli3 functions in controlling the proliferative expansion of distal progenitors are shared among dorsal and paired fins as well as tetrapod limbs. Mutant knockout *gli3* fins in medaka (*Oryzias latipes*) form multiple radials and rays, in a pattern reminiscent of the polydactyly observed in *Gli3*-null mutant mice. In limbs, *Gli3* controls both anterior–posterior patterning and cell proliferation, two processes that can be genetically uncoupled. In situ hybridization, quantification of proliferation markers, and analysis of regulatory regions reveal that in paired and dorsal fins, *gli3* plays a main role in controlling proliferation but not in patterning. Moreover, *gli3* down-regulation in *shh* mutant fins rescues fin loss in a manner similar to how *Gli3* deficiency restores digits in the limbs of *Shh* mutant mouse embryos. We hypothesize that the *Gli3/Shh* gene pathway preceded the origin of paired appendages and was originally involved in modulating cell proliferation. Accordingly, the distal regions of dorsal fins, paired fins, and limbs retain a deep regulatory and functional homology that predates the origin of paired appendages.

## Significance

In this study, we show that the inactivation of the *gli3* gene in medaka fish results in the formation of larger dorsal and paired fins. These mutant fins display multiple radial bones and fin rays which resemble polydactyly in *Gli3*-deficient mice. Our molecular and genetic analyses indicate that the size of fish fins is controlled by an ancient mechanism mediated by SHH-GLI signaling that appeared prior to the evolutionary appearance of paired fins. We also show that the key regulatory networks that mediate the expansion of digit progenitor cells in tetrapods were already in place in the fins of the last common ancestor between ray and lobe-finned fishes, suggesting an ancient similarity between distal fins and digits.

A fundamental issue in vertebrate biology is the relationship between fins and limbs. Limbs have a characteristic skeletal pattern of stylopod, zeugopod, and autopod, with a distal region composed of digits and mesopodial bones. While fossil taxa reveal intermediates in these conditions (1–3), the appendages of extant actinopterygians lack common features that allow comparison among the distal regions. Teleosts, for example, have fins with both endochondral and dermal bones. The distal fin typically has a series of cartilage elements with no obvious homology to digits or mesopodials, while rays develop as dermal, not endochondral, bones. Despite these dramatic differences in the distal anatomy of limbs and fins, the terminal region of fins reveals molecular similarities to limbs. Limbs have a characteristic pattern of two phases of expression of *Hox* genes, an early one that is associated with the specification of the stylopod and zeugopod and a later one associated with the formation of digits. Recent studies of the pattern of expression, function, and cell lineage of *Hox*-expressing cells in fish reveal that they have late-phase activity that is comparable to that of limbs (4). Lacking, however, is a knowledge of how deep these homologies extend in the tree of life. Are these kinds of similarities common to paired fins or are they a property of diverse paired and unpaired appendages of gnathostomes?

*Gli3*, a transcription factor expressed from early to late phases of limb development, has been shown to play multiple roles during limb morphogenesis. *Gli3* acts to set up the anterior–posterior (AP) axis of the limb bud and restricts *Shh* pathway activation to the posterior distal margin of appendages (5–8). This activity is mediated by the interactions of *Gli3* with the *Hand2* and *Hoxd* transcription factors. *Gli3*-deficient mice show *Shh* pathway derepression in the anterior limb bud margin, leading to the anterior expansion of the expression domains of posterior markers and concordant down-regulation of anterior transcription factors (9–14). Morphologically, these mutants reveal polydactylous manus and pes along with soft-tissue fusion of digits. Most importantly, mouse limb buds lacking both *Shh* and *Gli3* have identical gene expression patterns to those of *Gli3* single mutants and their limb skeletons are indistinguishable (12, 13). More recent studies have revealed that the roles played by *Gli3* in patterning and cellular proliferation during appendage morphogenesis can be genetically uncoupled. The control of the proliferative expansion of the autopod progenitors is hence key to constrain the number of digits to the pentadactyl pattern (15). While *Gli3* functions and interactions with *Shh* are essential features of limb development, little is known about its role in fins. This deficit is unfortunate because an understanding of this issue could reveal the origin of distal patterns between the two organs. To address this, we generated *gli3*-knockout (KO) mutants in medaka, a teleost fish with a single copy of *shh*, to assess gene expression, regulation, and ultimately evolution of the morphoregulatory mechanisms mediated by Gli3 in vertebrate appendages.

## Results

**Fig. 1.**
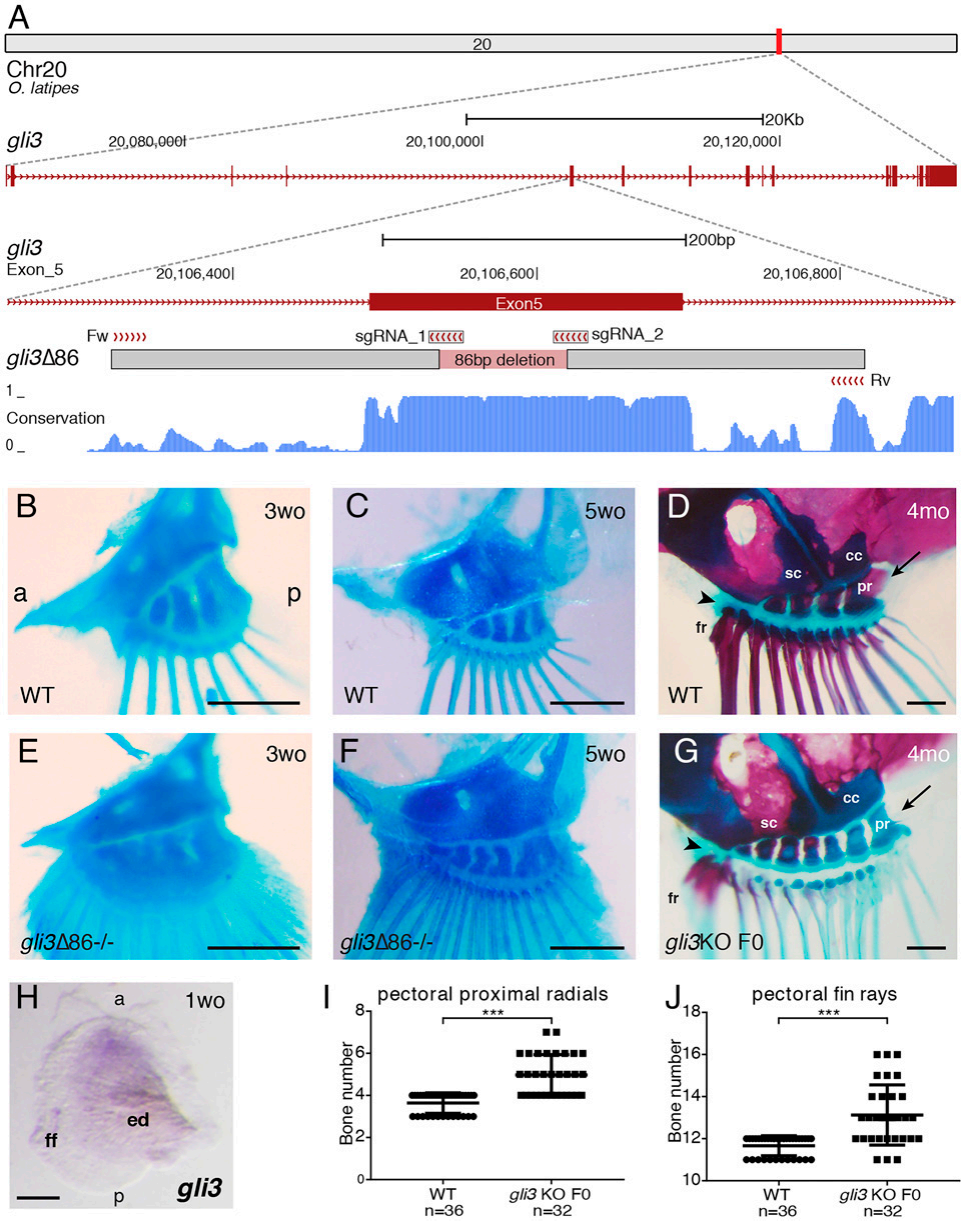
Medaka *gli3* mutants show an increased number of pectoral fin skeletal elements. (*A*) Stable lines harboring Δ86 deletions in medaka *gli3* exon 5 were generated by CRISPR-Cas9. The diagram shows the position of the deletion relative to the sgRNAs and primers used for screening along with the box of conservation with four fish species (stickleback, fugu, tetraodon, and zebrafish). The Δ86 deletion results in the generation of a premature STOP codon that truncates the predicted protein upstream of the zinc-finger DNA-binding domain of Gli3. (*B–G*) Alcian blue and Alizarin red staining of pectoral fin skeletons reveals a significantly increased number of proximal radial bones and fin rays in stable (*E* and *F*) and transient (*G*) *gli3* mutants, although major anatomical AP asymmetries appear unaltered. Note that, when compared with ones in the anterior margin, the two posterior proximal radials (pr) are larger in both *gli3* crispants and WT fish and articulate with the coracoid (cc) bone (arrows in *D* and *G*). In the anterior margin of the fin, both the rest of the proximal radials and the anterior-most fin rays (fr) articulate directly with the scapula (sc) in both WT and transient *gli3* mutants (arrowheads in *D* and *G*). (*B* and *C*) *n* ≥ 14 fins. (*E* and *F*) *n* ≥ 12 fins. (Scale bars, 250 μm.) mo, months-old; wo, weeks-old. (*H*) WT pectoral fin showing the expression of *gli3* in the anterior region of the developing pectoral fin bud (*n* = 10). (Scale bar, 100 μm.) ed, endochondral disk; ff, fin fold. (*I* and *J*) Quantification of skeletal elements in adult (4-mo-old) *gli3* crispants. Each point in the graphs represents the measurement of bone number in a single fin (*n* = 36 WT, *n* = 32 *gli3* KO F0). An unpaired *t* test was used for the statistical analysis of skeletal element number. ***P = 5.06 × 10^−10^ for the comparison between WT (mean 3.639) and *gli3* crispant (mean 4.969) pectoral proximal radial bones. ***P = 2.33 × 10^−7^ for the comparison between WT (mean 11.67) and *gli3* crispant (mean 13.13) pectoral fin rays. Bone-staining procedures in juvenile (*B*, *C*, *E*, and *F*) and adult fish (*D* and *G*) were performed in three independent experiments. a, anterior; p, posterior.

To explore the role of *gli3* in medaka fin patterning, we deployed CRISPR-Cas9 to disrupt its coding region via an 86-bp deletion in exon 5 (Fig. 1*A* and *SI Appendix*, Fig. S1). This mutation generates a frameshift that truncates the protein in the middle of the repressor domain. Adult fins could not be analyzed, as fish homozygous for this *gli3*-inactivated allele die between 2 and 5 wk of age, probably reflecting the pleiotropic functions of *gli3* in multiple tissues (*SI Appendix*, Fig. S1). By weeks 3 to 5, however, multiple anomalies were observed in the pectoral fin skeleton of *gli3* mutant escapers. In particular, these fins had an expanded number of radials and rays (Fig. 1 *B*, *C*, *E*, and *F*). Transient *gli3* mutants raised to adulthood displayed greatly expanded fins, with multiple supernumerary elements (Fig. 1 *D*, *G*, *I*, and *J*) in which mineralization appeared delayed (Fig. 1 *D* and *G*). Hence, *gli3*-deficient fish are similar to mouse and human *Gli3*/*GLI3* polydactylous mutants in having expanded appendages with extra bones. This skeletal analysis of pectoral fins also revealed that key morphological AP asymmetries appear unaltered in *gli3* mutants in comparison with wild-type (WT) controls (Fig. 1 *D* and *G*) (16).

**Fig. 2.**
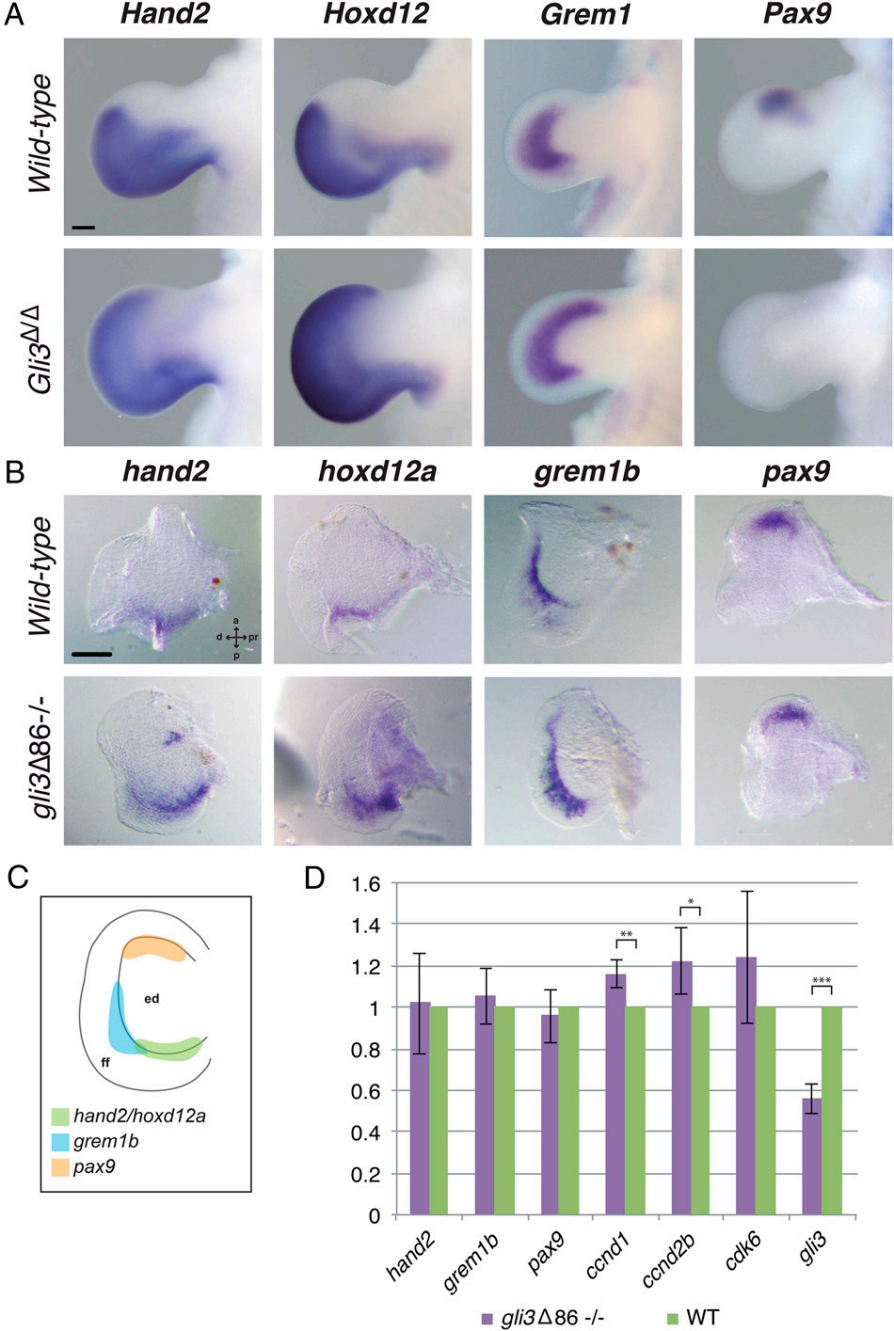
Expression of *gli3* target genes in WT and *gli3*-deficient pectoral fin buds. (*A*) *Hand2*, *Hoxd12*, and *Grem1* expression is anteriorly expanded and *Pax9* expression is lost in *Gli3*-deficient E11.5 mouse limb buds (*n* = 3 per marker and genotype). (Scale bar, 200 µm.) (*B*) ISH assays in WT and *gli3*Δ86^−/−^ medaka pectoral fins. At 6 dpf (stage 36), the inactivation of *gli3* does not greatly affect the expression pattern of *hand2*, *hoxd12a*, *grem1b*, or *pax9*, all genes involved in limb patterning (*n* ≥ 4 per marker and genotype). d, distal; pr, proximal. (Scale bar, 100 µm.) (*C*) Schematic representation of the *hand2*, *hoxd12a*, *grem1b*, and *pax9* expression domains in 6-dpf WT medaka pectoral fins. Note that the *grem1b* expression domain (shown in *B*) runs along the margin of the endochondral disk (ed) and extends into the fin fold. (*D*) Gene expression quantification by qPCR in WT and *gli3*Δ86^−/−^ medaka pectoral fins at 11 dpf. The relative expression of the proliferation regulators *ccnd1* and *ccnd2b* is significantly increased in mutant fins. Mutant values (purple bars) are normalized against WT values (green bars), and represented as mean ± SD. Note that the *gli3*Δ86 allele is still transcribed in homozygous mutant fins, although at lower levels due to nonsense-mediated mRNA decay. *n_hand2_* = 4, *n_grem1b_* = 3, *n_pax9_* = 3, *n_ccnd1_* = 2, *n_ccnd2b_* = 4, *n_cdk6_* = 3, *n_gli3_* = 4; ***P_gli3_ = 9.026 × 10^−9^, **P_ccnd1_ = 0.0022, *P ≤ 0.05, *P_ccnd2b_* = 0.011.

Next, we examined the expression profiles of genes involved in appendage patterning, in particular those known in tetrapods to interact with *Gli3* during limb bud outgrowth. Similar to tetrapod limbs, *gli3* expression in medaka is higher in the anterior margin of the pectoral fin bud, while *shh* remains restricted to the posterior-most mesenchyme (Fig. 1*H* and *SI Appendix*, Fig. S2). In mouse limb buds, *Gli3* inactivation causes the anterior expansion of posterior markers such as *Hand2*, 5'*Hoxd*, and *Grem1* and down-regulation of anteriorly expressed genes (e.g., *Pax9*; Fig. 2*A*) (9, 10, 12–14). In contrast, in situ hybridization (ISH) analysis in medaka *gli3* mutant pectoral fins at stage 36 (6 d postfertilization [dpf]) revealed no obvious changes in the expression domains of these genes, as their expression patterns appear largely similar to those of WT embryos (Fig. 2*B*). At later stages (8 dpf), we noted that, while *hoxd12a* expression was low in both WT and *gli3* mutants, there was an evident expansion of the *grem1b* expression domain along the rim of the endochondral disk in *gli3*-deficient pectoral fin buds (*SI Appendix*, Fig. S3). Because *Gli3* has also been shown to control the proliferative expansion of mouse autopod progenitors through the direct transcriptional modulation of cell-cycle regulators (15), we next used qRT-PCR to assess the expression levels of *gli3*-*shh* proliferative targets in fin buds (Fig. 2*D*). This analysis revealed the up-regulation of *ccnd1* and *ccnd2* in *gli3*-deficient fins, uncovering a role for Gli3 in controlling distal proliferation during fin outgrowth. Both the deregulation of G1–S cell-cycle modulators and of *Grem1* are features observed upon the late, conditional inactivation of *Gli3* in mouse handplate progenitors, which develop polydactyly in the absence of AP polarization defects (15).

Our morphological, gene expression, and qPCR analyses point toward a role for *gli3* in cell proliferation, rather than AP patterning, during pectoral fin development. To explore this hypothesis and reveal its phylogenetic distribution, we analyzed Gli3 chromatin immunoprecipitation sequencing (ChIP-seq) data from mouse limb buds. This strategy revealed that *Gli3* preferentially binds at the promoters of these proliferation genes (*SI Appendix*, Fig. S4). These regulatory regions are present in diverse tetrapods and fishes, revealing their phylogenetic generality. In contrast, *Gli3* regulation of patterning genes (e.g., *Grem1*, *Hand2*, *Pax9*, and *Hoxd* genes) takes place mainly through distal *cis*-regulatory elements (CREs) that are mostly not conserved in fish (17, 18). In particular, most of the Gli3-binding CREs previously reported within the regulatory landscapes of *Hand2* and *Hoxd* genes appear to be tetrapod innovations (*SI Appendix*, Figs. S4 and S5) while, of the two characterized *Grem1* enhancers bound by Gli3, only one [GRS1 (18)] is deeply conserved in all vertebrates, and could mediate the late AP expansion of *grem1* in medaka *gli3* mutant fins (*SI Appendix*, Fig. S4). Overall, these data support the notion that the incorporation of *Gli3* in early patterning events in appendages is a more recent novelty within derived gnathostomes that evolved through the progressive acquisition of new distal CREs.

**Fig. 3.**
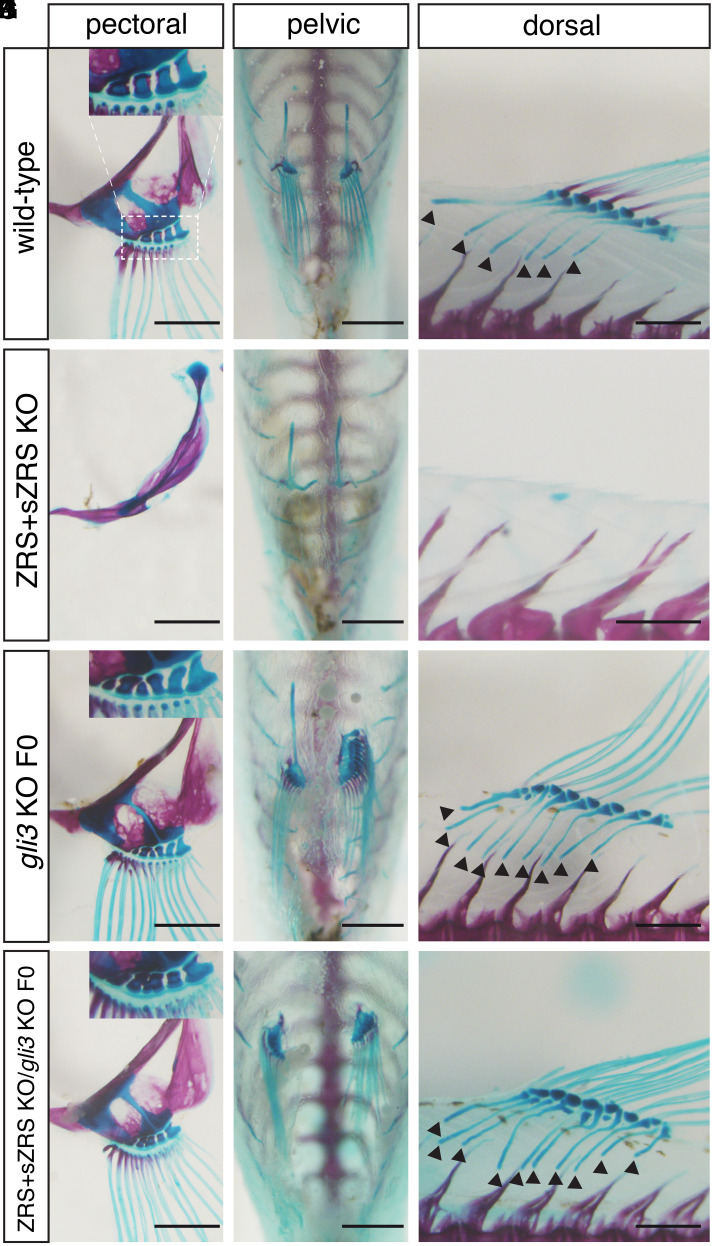
Mosaic inactivation of *gli3* in fins lacking *shh* completely rescues the formation of dorsal, pelvic, and pectoral fins. Skeletal staining and fin morphology in adult fish. (*A–F*) In contrast to WT (*A–C*), all fin elements are absent in the dorsal, pelvic, and pectoral fins from ZRS+sZRS mutant fish (*D–F*). (*G–I*) CRISPR-Cas9 disruption of *gli3* significantly increases the number of pectoral and dorsal fin bone elements (black arrowheads in *I*) compared with WT (*A–C*). (*J–L*) *gli3* down-regulation in the homozygous ZRS+sZRS *shh* mutant background totally rescues the dorsal, pelvic, and pectoral fin phenotypes. Note that there is a delay in the ossification of dorsal fin rays in *gli3* single and *gli3/ZRS+sZRS* compound mutants. (Scale bars, 1 mm.) Microinjection of the *gli3* CRISPR mixture and bone-staining procedures were performed in three independent experiments.

A main function of *Shh* signaling is to antagonize the constitutive proteolytic processing of Gli3 to its transcriptional repressor isoform (19). Genetic analysis in mice has shown that *Gli3* deficiency is able to rescue the loss of distal limb bones observed in *Shh*-null embryos, leading to polydactyly that is identical to that observed upon *Gli3* inactivation alone (12, 13). To examine this relationship in medaka, we produced *gli3*/*shh* double mutants transiently, as *gli3*-deficient fish are not viable. Transient inactivation of *gli3* in a ZRS+sZRS *shh* mutant background (20) is sufficient to rescue the *shh* loss-of-function phenotype (agenesis of pectoral, pelvic, and dorsal fins). Analogous to the genetic interaction observed in mutant mouse autopods, the fin skeleton of ZRS+sZRS/*gli3* F0 double mutants resembles those of *gli3* crispants. This effect was seen in both paired and unpaired fins, as supernumerary bones were seen in pectoral, pelvic, and dorsal fins (Fig. 3 and *SI Appendix*, Fig. S6). Interestingly, as observed previously in the ZRS+sZRS *shh* mutant (20), the anal fin is not affected by any of these mutant conditions (*SI Appendix*, Fig. S7).

## Discussion

Altogether, our results show that the presence of the *shh/gli3* regulatory network in fish fins, so vital for limb formation and digit patterning, is primitive to limbs. Moreover, its functions in unpaired dorsal fins, widely recognized precursors of paired appendages, suggest that the recruitment of this network may have preceded the origin of paired fins themselves.

The correlation of expanded radials and rays in *gli3* fin mutants with the polydactyly in mouse *Gli3* mutants points to a deep homology among the distal tissues of gnathostome appendages. Our analyses suggest that the primitive function of the *Shh/Gli3* module in appendages was to control the proliferative expansion of the distal mesenchyme. Interestingly, the transcriptional control of cell-cycle effectors is an ancient feature of HH signaling, as Ci, the fly ortholog of *Gli3*, also directly regulates several *cyclin* genes (21, 22). In contrast, the fully wired AP patterning systems controlled by *Gli3* likely evolved later during evolution, probably in the tetrapod lineage through the appearance of novel far-acting *cis*-regulatory regions (23, 24). As expected, given their dependence on SHH signaling for their posterior up-regulation (25–28), some deeply conserved Gli-binding CREs are present in the *Hoxd* and *Grem1* genomic landscapes. Moreover, our observation that the *grem1b* expression domain is expanded in *gli3*-deficient fin buds at 8 dpf suggests that some aspects of *grem1b* regulation are controlled by Gli3 at these advanced stages. This is highly reminiscent of the situation in the mouse, as failure to terminate *Grem1* expression in the anterior margin of the handplate leads to a delay in chondrogenic differentiation that contributes to the polydactyly observed in *Gli3*-deficient limb buds (15). The highly conserved GRS1 enhancer is a good candidate to implement some aspects of Gli3-mediated repression of *Grem1*/*grem1b* at these late stages of appendage development, as its activity is sensitive to *Gli3* gene dosage in the mouse (18). Finally, another possibility explaining the lack of apparent polarization defects in medaka *gli3* mutants would be that SHH-mediated AP patterning in early teleost fins (25, 26) is mediated in a *gli3*-independent manner by a Gli2 factor(s), capable of being processed into GLI activator and repressor isoforms (29, 30). Interestingly, *Gli2* has been genetically shown to cooperate with *Gli3* in providing posterior identity to the mouse autopod (31).

Overall, our results imply that the distal regions of appendages have a common evolutionary origin and that the *Shh/Gli3* network was modified in fish and tetrapod lineages to produce fin radials and rays in the former and digits in the latter. Interestingly, the only appendage that does not follow these rules, the anal fin, also has anomalous patterns of *shh* regulation. The absence of these networks in anal fins points to a separate evolutionary origin for anal fins, presumably by independent cooption of fin patterning networks in a novel site.

## Materials and Methods

### Animal Experimentation.

All experiments involving fish and mice performed in this work conform to European Community standards for the use of animals in experimentation and were approved by the ethical committees from the Universidad Pablo de Olavide, Universidad Mayor, and Consejo Superior de Investigaciones Científicas and the Andalusian government.

### Fish Stocks.

Medaka WT (iCab) and ZRS+sZRS KO [Δ3.4kb (20)] strains were maintained and bred under standard conditions (32). Embryos were staged in hours postfertilization as previously described (33). Medaka *gli3*_Δ86 and ZRS_Δ3.4kbs mutant alleles were maintained in heterozygosis due to the higher lethality of the homozygous mutants. *gli3*_Δ86 homozygous null larvae died between 2 and 5 wk of age.

### Skeletal Staining.

Alcian blue and Alizarin red staining experiments were performed as previously described (20, 34). In brief, fish were fixed with 10% neutral-buffered formalin overnight or longer. After several washes with deionized water, cartilage was stained overnight using a 0.1% Alcian blue solution (Alcian blue 8GX; PanReac AppliChem) in 30% acetic acid and 70% ethanol. Fish were then washed with deionized water and changed to a solution containing 1% trypsin from bovine pancreas (PanReac AppliChem) and 30% saturated sodium borate for 8 h (or longer) with gentle shaking at room temperature. After trypsin enzyme treatment, specimens were rinsed several times with deionized water and transferred to 0.5% aqueous KOH solution. Fish bones were finally stained overnight with a solution containing saturated Alizarin red S (PanReac AppliChem) in 0.5% KOH. After several washes with a 0.5% KOH solution, fish were gradually transferred to glycerol for documentation. Specimens were visualized with an Olympus SZX16 binocular microscope and photographed with an Olympus DP71 camera.

### CRISPR-Cas9 Design and *gli3* Mutant Generation.

Two single guide RNAs (sgRNAs) targeting exon 5 of medaka *gli3* were designed using the CRISPRscan (35) and CCTop (36) CRISPR design online tools. sgRNAs were generated and purified for injection as previously described (37). For sgRNA generation, the following primers were aligned (by PCR) to a universal CRISPR primer: *gli3* exon 5 sgRNA1: 5'-taatacgactcactataGGGCGGATGTAGTCCATGTAgttttagagctagaa-3' and *gli3* exon 5 sgRNA2: 5'-taatacgactcactataGGGGTGAGATCCGAATGAGGgttttagagctagaa-3' (in both primers the target site is identified by capital letters). Following synthesis, 5 nL of a solution containing both sgRNAs at a concentration of 40 ng/μL and Cas9 protein (Addgene; 47327) at a concentration of 250 ng/μL (38) were injected into one-cell-stage medaka embryos. Oligos used for screening of genomic DNA deletions flanking CRISPR target sites were the following: forward primer 5'-CGTGAGTTTCACAGCAACAATTA-3' and reverse primer 5'-CAGCCTCACTGATCAATTTCAG-3'. Mutations in *gli3* were analyzed by standard PCR and gel electrophoresis as the distance between both sgRNA protospacer adjacent motif (PAM) sequences was 82 bp, long enough to create a deletion easily detected by a shift in the PCR band. Specific deletions in the *gli3* gene were further analyzed by Sanger sequencing of the PCR product from F1 embryos (Stab Vida).

### Statistical Analyses.

The number of pectoral fin proximal radial bones, pectoral fin rays, and dorsal proximal pterygiophores was manually counted in adult WT and *gli3* crispant fish after a bone-staining procedure. Differences in the number of skeletal elements between both groups were tested by applying an unpaired *t* test using GraphPad Prism software. Paired two-tailed *t* tests were used for the statistical analysis of average differences in gene expression levels between mutant and WT samples as measured by qRT-PCR.

### Medaka In Situ Hybridization.

Depending on the genomic location of the designed primers, antisense digoxigenin-labeled RNA probes were prepared from 4-dpf medaka complementary DNA (cDNA) or gDNA (*SI Appendix*, Table S1). *shh* (20), *hand2*, *hoxd12a*, *grem1b*, and *pax9* RNA probes were synthesized by cloning the DNA-amplified region using the StrataClone PCR Cloning Kit (240205-5; Agilent Technologies; *hoxd12a*) or pGEM-T Easy Vector (A1360; Promega; *pax9*, *grem1b*, and *hand2*), and these linearized vectors were used as templates for RNA transcription. The *gli3* probe was directly transcribed from the amplified DNA since the SP6-RNA polymerase promoter sequence (labeled red in *SI Appendix*, Table S1) was included in the primers used for amplicon amplification.

Heterozygous animals were mated in order to collect embryos to perform ISH assays. The embryos were maintained at 28 °C in E3 medium (5 mM NaCl, 0.17 mM KCl, 0.4 mM CaCl_2_, 0.16 mM MgSO_4_, and 0.00003% methylene blue) until 4, 6, 8, and 11 dpf and fixed in 4% paraformaldehyde/phosphate-buffered saline (PBS) for 48 h at 4 °C. Embryos were manually dechorionated when needed (stages 4 to 8 dpf), dehydrated through an increasing MeOH washing series, and kept at −20 °C until the experiments were performed. Overall, the specimens were prepared, hybridized (2 ng probe per microliter), and stained as previously described (39, 40). Embryos at stages older than 4 dpf were permeabilized with 10 µg of ProtK for 7 min.

In order to analyze the expression of the different genes in mutant and WT siblings, samples were genotyped after ISH assays. gDNA was extracted using Chelex 100 sodium form (C7901; Sigma) from a piece of tail dissected from each individual larva and standard PCR reactions were performed with primers flanking the deleted region (Fig. 1 and *SI Appendix*, Table S1). Subsequently, gene expression patterns in homozygous null and WT pectoral fins were analyzed and documented. Both pectoral fins from each genotyped larva were dissected and transferred to a drop of 3% methyl cellulose (M0387; Sigma-Aldrich) on a slide for documentation using an Olympus SZX16 (model SZX2-ILLB) binocular scope and an Olympus DP71 camera.

### Quantitation of Transcript Levels in Medaka Fins by qRT-PCR.

Larvae were raised as described above until 11 dpf and deeply anesthetized with 160 mg/L of tricaine (ethyl 3-aminobenzoate methanesulfonate salt; MS-222; Merck) before dissecting their pectoral fins. The dissection was performed in a drop of embryos’ medium and the fins were rapidly moved to an ice-cold drop of PBS. Every pair of dissected pectoral fins coming from a single larva was transferred to a separate 1.5-mL tube containing 50 µL TRIsure (BIO-38032; Bioline) and stored at −20 °C until larvae were genotyped (*Medaka In Situ Hybridization*). In order to obtain sufficient RNA material, each biological replicate consisted of 20 pairs of pectoral fin buds per genotype (WT and *gli3*-deficient). RNA was extracted following the TRIsure manufacturer’s instructions and equivalent amounts of mutant and WT RNA were used to synthesize cDNA using the High Capacity cDNA Reverse Transcription Kit (Applied Biosystems–Thermo Fisher Scientific; 4368814). The expression levels of medaka *hand2*, *grem1b*, *pax9*, *ccnd1*, *ccnd2b*, *cdk6*, and *gli3* in the developing fins were quantified through qRT-PCR (CFX96 Real-Time C1000 thermal cycler; Bio-Rad) and normalized to the expression level of the housekeeping gene *ef1a* (*SI Appendix*, Table S1). qPCR reactions were performed in triplicate from two to four biological replicates using iTaq Universal SYBR Green Supermix (Bio-Rad; 172-5124; *n_hand2_* = 4, *n_grem1b_* = 3, *n_pax9_* = 3, *n_ccnd1_* = 2, *n_ccnd2b_* = 4, *n_cdk6_* = 3, *n_gli3_* = 4). The expression levels in mutant samples were calculated in relation to WT controls (average set to 100%). Assuming a normal distribution of the data, a paired two-tailed *t* test was performed to test the significance of differences among sample averages. As the *gli3*Δ86 mutation induces a frameshift that results in premature stop codons, *gli3* transcription levels are strongly reduced in *gli3*Δ86 homozygous fins due to nonsense-mediated messenger RNA (mRNA) decay.

### Mouse Experiments.

Embryonic day 11.5 (E11.5) (44 to 48 somites) *Gli3*-deficient and WT embryos were processed for ISH with riboprobes recognizing *Hand2*, *Hoxd12*, *Grem1*, and *Pax9* transcripts (*n* = 3 per marker and genotype), as previously described (15). Gli3 ChIP-seq and assay for transposase-accessible chromatin with sequencing (ATAC-seq) data generated from E10.5 mouse limb buds have been previously published (41, 42).

## Data Availability

All study data are included in the article and/or *SI Appendix*. Previously published data were used for this work (PMID: 31989924; PMID: 32268095). **Note Added in Proof.** During the review of this manuscript, another study (43) reported an additional highly-conserved Grem1 enhancer, termed CRM5, that is, as GRS1 (renamed as CRM2), also regulated by Gli3.
